# Delivering Virtual Group Interventions for Youth With Persisting Symptoms After Concussion (Move&Connect-Youth) and Their Caregivers (Move&Connect-Caregivers): Fidelity and Engagement Evaluation

**DOI:** 10.2196/88771

**Published:** 2026-09-21

**Authors:** Andrea Hickling, Hiba Al-Hakeem, Andrew Lovell, Sophie Madge-Nyren, Kylie D Mallory, Jennifer L Ryan, Christine F Provvidenza, Ruth Wilcock, Patricia Knapp, Brenda Knapp, Shari L Wade, Keith Owen Yeates, Shannon E Scratch

**Affiliations:** 1 Bloorview Research Institute, Holland Bloorview Kids Rehabilitation Hospital, 150 Kilgour Rd, Toronto, ON, M4G1R8, Canada, 1 416-425-6220; 2 Department of Occupational Science and Occupational Therapy, University of Toronto, Toronto, ON, Canada; 3 Department of Psychology, University of Windsor, Windsor, ON, Canada; 4 Ontario Brain Injury Association, Thorold, ON, Canada; 5 Division of Rehabilitation Medicine, Cincinnati Children's Hospital Medical Center, Cincinnati, OH, United States; 6 Department of Pediatrics, University of Cincinnati, Cincinnati, OH, United States; 7 Department of Psychology, Alberta Children’s Hospital Research Institute, University of Calgary, Calgary, AB, Canada; 8 Rehabilitation Sciences Institute, Temerty Faculty of Medicine, University of Toronto, Toronto, ON, Canada; 9 Department of Pediatrics, Temerty Faculty of Medicine, University of Toronto, Toronto, ON, Canada

**Keywords:** concussion, brain injury, telehealth, telemedicine, virtual care, rehabilitation, pediatrics, parents, group intervention, qualitative

## Abstract

**Background:**

Youth with persisting symptoms after concussion (PSaC) can experience ongoing physical, cognitive, and emotional challenges, and their caregivers experience increased stress. There are currently limited interventions that support youth with PSaC or their caregivers. *Move&Connect* is a virtual group program with 2 parallel interventions: *Move&Connect-Youth* (*M&C-Y*) and *Move&Connect-Caregivers* (*M&C-C*). *M&C-Y* includes concussion education, active rehabilitation, and goal setting. *M&C-C* uses psychoeducation and practical tools to promote family communication and problem solving.

**Objective:**

Within the context of a broader pilot study of *M&C-Y* and *M&C-C*, we completed a fidelity and engagement evaluation with the objectives of (1) evaluating intervention fidelity and (2) exploring facilitators and barriers to youth and caregiver engagement.

**Methods:**

*M&C-Y* and *M&C-C* were delivered virtually using Zoom Healthcare and ran in parallel. Youth with PSaC and caregivers were recruited from a children’s rehabilitation hospital in Ontario, Canada. Intervention fidelity was evaluated according to the 5 principles of fidelity from Bellg et al: design, training, delivery, receipt, and enactment. Intervention delivery, attendance, and engagement ratings were monitored through weekly logs and field notes. Participants completed exit satisfaction surveys, and a subset of youth and caregivers completed semistructured interviews to understand facilitators and barriers to engagement. Descriptive statistics were used to analyze logs and surveys. Directed content analysis, guided by a motivational framework, and inductive coding to identify barriers and facilitators to engagement were applied to qualitative data.

**Results:**

A total of 34 youth (age: mean 15.1, SD 2.28 years) and 36 caregivers (age: mean 48.9, SD 5.92 years) participated in 10 *M&C-Y* and 9 *M&C-C* groups, respectively. Most youth identified as girls (24/34, 71%), and most caregivers identified as mothers (30/36, 83%). Clinician facilitators adhered to the components of the intervention protocols between 92% and 97% of the time in *M&C-Y* and 82% and 93% of the time in *M&C-C*. The majority of youth (22/34, 65%) and caregivers (24/36, 67%) attended 5 or more sessions. In addition, 82% (28/34) of youth and 94% (34/36) of caregivers agreed they would recommend the interventions to others. Qualitative findings revealed youth engagement was supported by motivation, comfort in the virtual setting, interactive intervention components, feelings of trust, and diverse and modifiable exercises. Barriers to youth engagement included ongoing symptoms, discrepancies between exercises and physical abilities, and previous knowledge of education. Caregiver engagement was facilitated by perceived benefits of *M&C-C*, emotional connection and validation, and comfort and convenience of the virtual setting. Barriers to caregiver engagement included stress, competing scheduling demands, and limited reminders or access to intervention materials.

**Conclusions:**

The pilot study of *M&C-Y* and *M&C-C* was conducted with high delivery adherence. Exit satisfaction surveys combined with qualitative data revealed high levels of caregiver satisfaction and engagement, whereas youth satisfaction and engagement had some variability, particularly related to the exercise. Findings demonstrate important implications for delivering virtual group interventions for youth with PSaC and their caregivers.

## Introduction

### Background

After a concussion, youth can experience an array of physical, cognitive, and emotional and behavioral symptoms [1]. Although most youth recover relatively quickly from concussion, up to 30% continue to experience persisting symptoms after concussion (PSaC) for months or even years [2,3]. PSaC can negatively impact youths’ quality of life [4,5] and lead to ongoing mental health challenges [6,7]. Youth with PSaC also experience feelings of isolation and a lack of social support and understanding from peers [8].

An interdisciplinary approach is recommended to support youth with PSaC [9,10], and active rehabilitation (ie, subthreshold aerobic exercise, coordination exercises) [11] and psychoeducation are considered standard best practices in the treatment of PSaC [12]. Active rehabilitation has been shown to reduce symptoms and improve mental health outcomes in youth with PSaC [13-16]. Psychoeducational intervention about recovery expectations and treatment recommendations is important for youth with PSaC and their caregivers [17,18]. In addition to these approaches, emerging research has explored the feasibility and impact of providing concussion care in a virtual model [19-21] and virtual active rehabilitation to youth with PSaC [22-25]. These virtual approaches are safe and tolerable for youth with PSaC and provide comfort and convenience for families [19,21-23,26].

Caregivers play a critical role in managing their child’s PSaC and describe burden [27] and elevated stress regarding their child’s recovery [28]. Caregiver and family functioning may have an important bidirectional association with PSaC after pediatric concussion [29]. Providing caregivers with psychoeducation and therapeutic support can help to navigate challenges after concussion [30]. Caregivers report a need for clear and reliable information about concussion recovery [31], and caregiver education on concussion signs and symptoms has been linked to reduced recurrence rates [32]. Despite this, there is a dearth of interventions tailored to caregivers of youth with PSaC and a clear need to develop support that targets caregivers’ well-being during concussion recovery [33,34].

### Move&Connect

The *Move&Connect* program was designed to support youth with PSaC and their caregivers and consists of 2 parallel intervention arms: *Move&Connect-Youth* (*M&C-Y*: for youth with PSaC) and *Move&Connect-Caregivers* (*M&C-C*: for caregivers of youth with PSaC). *M&C-Y* and *M&C-C* are 6-week virtual group interventions with 1-hour sessions that were developed using the intervention mapping framework [35] and codesigned with youth, caregivers, and clinicians. The detailed design and development processes of these interventions are described elsewhere [36,37].

Delivering rehabilitation interventions such as *M&C-Y* and *M&C-C* via virtual platforms (eg, Zoom Healthcare) can enhance access to services, support participation, and promote continuity of care [38,39]. The virtual group model in *Move&Connect* is particularly appropriate for youth with PSaC and their caregivers due to geographic inequities accessing specialized concussion care [40-42], family burden navigating appointments [43,44], and engagement of individuals who may become symptomatic with travel or in-person appointments [42]. The *Move&Connect* program differs from existing virtual concussion and group rehabilitation programs [25,45-48] due to the parallel youth and caregiver intervention design, theory driven development, and interdisciplinary delivery.

*M&C-Y* is guided by the biopsychosocial model [49] and includes psychoeducation, active rehabilitation, and goal-setting. Each week, the intervention begins with an ice breaker followed by an education or discussion topic (eg, headache management, managing stress, advocacy). Youth then complete exercises (ie, cardiovascular, balance, coordination, strength) with the goal of improving their confidence and tolerance to different physical demands. All exercises are designed to be completed at home with no equipment required. In-session music during exercise and weekly polls are used to engage participants. At the end of the session, youth set a weekly goal, often related to the psychoeducation topic or exercise, which is reviewed and discussed in a solution-focused manner [50] the following week [36].

*M&C-C* is guided by the family-directed approach to brain injury (FAB) model [51] and includes psychoeducation and active skill development to promote family communication and problem-solving. Psychoeducation, discussion, and activities are completed as a group and are related to weekly themes: the ripple effect, school advocacy, considering your child’s well-being, family and you, stress and daily challenges, and parenting is hard. Weekly take-home reflection activities are introduced at the end of each session, and caregivers share their insights during the group discussion the following week [37].

Initial testing of *M&C-Y* [36] (n=10) and *M&C-C* [37] (n=9) revealed that the interventions were feasible based on the key domain areas (recruitment, acceptability, and evaluation of participant responses) from Orsmond and Cohn [52] and our a priori criteria. Youth indicated that *M&C-Y* was enjoyable and they were able to form meaningful peer connections [36]. Caregivers indicated that *M&C-C* was a safe space to share personal experiences and they valued receiving advice from facilitators and their peers [37].

### Intervention Fidelity and Engagement

Following establishment of feasibility, it is important to consider intervention fidelity, which is the ongoing assessment, monitoring, and enactment of the reliability and internal validity of a study [53]. Intervention or treatment fidelity (herein referred to as intervention fidelity) is a critical part of intervention research as it increases confidence that changes in the study outcome of interest (dependent variable) are due to the intervention (independent variable) [54]. A rigorous fidelity plan should include study design, training, and quality and consistency of delivery [54,55]. Importantly, recent studies have emphasized moving beyond only assessing intervention delivery to including a greater focus on the understanding of participants’ receipt and enactment (collectively called engagement) of intervention activities [53,56,57]. Understanding intervention engagement is especially important as it is multifaceted and may be influenced by numerous contextual factors [57] (eg, virtual delivery, group setting, multiple intervention components). Engagement has previously been described by King and colleagues [58] as the affective (expecting positive outcomes), cognitive (believing that therapy will be effective), and behavioral (actively participating in therapy tasks) involvement or investment in a therapy session [58]. Using engagement-promoting strategies is recommended to improve adherence to interventions [59-62]. Additionally, family members being actively engaged in their child’s rehabilitation journey is associated with better outcomes [63]. Thus, exploring intervention fidelity and participant engagement are vital steps in delivering an intervention study.

### Objectives

Based on the aforementioned promising feasibility findings, we conducted a pilot study of *M&C-Y* and *M&C-C*. Within the context of the broader pilot study, the goal of this paper was to (1) evaluate intervention fidelity and (2) explore facilitators and barriers to youth and caregiver engagement.

## Methods

### Study Design

This paper describes a multimethod fidelity and engagement evaluation of *M&C-Y* and *M&C-C*. Between November 2022 and June 2025, 10 *M&C-Y* and 9 *M&C-C* groups were delivered. Group interventions were delivered virtually using Zoom Healthcare. Two trained clinician facilitators delivered each *M&C-Y* (occupational therapists and/or physiotherapists) and *M&C-C* (neuropsychologist, neuropsychology trainee, and/or social worker) session. A research staff observer attended all *M&C-Y* and *M&C-C* sessions to record observations and support any technological challenges. Youth were allocated to groups by age/grade, when possible (ie, elementary and high school), to account for developmental differences. Caregivers were also allocated to groups for those with elementary or high school–aged children. Participants were provided with weekly reminder emails of upcoming sessions including any supplies and weekly handouts or materials in advance of each session.

To ensure safety and personalization during *M&C-Y*, modifications to make each exercise more or less challenging were provided, breaks were encouraged, and clinicians monitored participant symptom changes. Consistent with concussion best practice guidelines [12], if youth experienced >2-point change in their symptoms on a symptom severity scale ranging from 0 to 10, they were instructed to stop the activity until their symptoms had returned to their baseline level. Virtual breakout rooms were utilized for 1-on-1 conversations regarding symptom monitoring. Reminders about participant privacy and group confidentiality were provided at the beginning of each session. The study’s principal investigator randomly audited intervention sessions. No adverse events occurred during the study.

Youth and caregiver participants also completed outcome measures before (T_0_), immediately following (T_1_), and 3 months following (T_2_) intervention completion. Clinical outcome results will be described in subsequent publications, as the primary contribution of this paper is the evaluation of intervention fidelity and engagement. Intervention fidelity evaluation involved data collected during intervention delivery and at the T_1_ follow-up. Engagement evaluation involved optional youth and caregiver exit interviews completed at T_1_. The qualitative components of the study follow guidelines outlined in the Consolidated Criteria for Reporting Qualitative Studies (COREQ) checklist [64] (see Checklist 1).

### Recruitment and Eligibility Criteria

Youth were included if they were between the ages of 8 years and 21 years, had a diagnosis of concussion, and had been experiencing PSaC for >4 weeks at intervention onset. Youth were excluded if they had a physical condition that would impact participation (eg, requiring a mobility device), a disorder of social cognition or communication (eg, intellectual disability, autism spectrum disorder), a functional neurological disorder (eg, conversion disorder, somatization), or an acute psychiatric condition that resulted in a recent hospital admission. Given the pilot nature of the study, youth with these conditions were excluded as they may have unique support and safety needs. Caregivers were included if they had a child (aged 8‐21 years) who was diagnosed with a concussion and had been experiencing PSaC for >4 weeks at intervention onset. Youth and caregivers had to be able to read and understand English, be willing to engage in weekly sessions, and have access to a reliable internet connection. Youth and caregivers could enroll as a dyad (ie, both youth and caregiver enroll) or independently (ie, youth could enroll without caregiver; caregiver could enroll without youth). Recruitment predominantly occurred via convenience sampling through concussion services at Holland Bloorview Kids Rehabilitation Hospital (HBKRH) [10], but community participants were also eligible.

### Ethical Considerations

Ethics approval was obtained from HBKRH (REB #0513). All procedures complied with the ethical principles outlined in the Declaration of Helsinki. Youth and caregivers were informed that their decision to participate in the study would not affect the care that they received at HBKRH. A capacity assessment (ie, a formal assessment of decision-making capacity) [65] was performed for all youth participants, and all participants (youth and caregivers) provided informed written consent.

### Study Measures

#### Intervention Fidelity Assessment

This pilot study used the framework by Bellg et al [55] for assessing the fidelity of the *M&C-Y* and *M&C-C* interventions, which describes 5 key domains to ensure optimal intervention fidelity: (1) intervention design, (2) provider training, (3) intervention delivery, (4) intervention receipt, and (5) intervention enactment. Rather than define strict a priori fidelity thresholds, we used triangulation to evaluate intervention delivery, receipt, and enactment. Multiple data sources and approaches were used for each domain to allow for triangulation and reduction of potential bias [57] (Table 1).

**Table 1.** Intervention fidelity components and assessment approaches. Table adapted from Ginsburg and colleagues [57].

Intervention fidelity component [55]	Our approach to fidelity assessment
Fidelity of design	
Intervention is defined and operationalized consistent with its underlying theory	Intervention approach and components designed with incorporation of underlying theory; see Scratch et al [36] and Al-Hakeem et al [37] for details
Fidelity of training	
Clinician facilitator training	Research and institutional orientation Clinicians provided with intervention manuals and materials Train-the-trainer approach including session shadowing and discipline specific peer debriefing Feedback provided by trainees to inform train-the-trainer approach
Fidelity of delivery	
Adherence to protocol (components, timing) and consistent delivery	Clinician delivery checklist completed after each session Debriefing after each session with clinicians and research staff observer Bi-weekly research team meetings Clinician consultation with study principal investigator, as needed
Fidelity of receipt	
Participants understood/can use intervention components	Participant attendance tracking Research staff observer field notes
Fidelity of enactment	
* *Engagement in Move&Connect activities	Participant engagement rating Exit satisfaction survey Research staff observer field notes Semistructured interviews^a^

a Used in objective 2 to further explore barriers and facilitators to engagement.

#### Intervention Fidelity Tools

All fidelity tools, with the exception of the semistructured interviews, were captured using REDcap [66].

##### Clinician Delivery Checklist

To monitor adherence to the components and timing of the intervention delivery, 2 clinician facilitators completed the clinician delivery checklist weekly after each session. The *M&C-Y* clinician delivery checklist asked if the education, exercise, and goal setting components of the program were delivered as intended (3 yes/no questions). The *M&C-C* clinician delivery checklist asked if the education, activity, and take-home reflections were delivered as intended (3 yes/no questions). An open-ended text box allowed clinicians to explain any deviations from intervention delivery or additional contextual information.

##### Attendance Tracking Log

Youth and caregiver attendance for each session was monitored in an attendance tracking log completed by the research staff observer. The attendance tracking log contained information regarding session number (1-6), attendance (yes/no), and if a participant was late for the session (yes/no). An open-ended text box allowed for any additional contextual information related to attendance (eg, if a participant emailed that they were unable to attend).

##### Research Staff Observer Field Notes

A structured field note template was used by the research staff observer in all *M&C-Y* and *M&C-C* sessions to capture behavioral observations, participant goals, and any feedback on the intervention provided by participants throughout the sessions.

##### Participant Engagement Rating

The research staff observer completed the participant engagement rating for each participant that attended each *M&C-Y* and *M&C-C* session. The participant engagement rating was developed by the research team to provide an exploratory measure of participation and engagement and was based on insights and observations from initial feasibility testing [36]. The 1‐3 rating asked, “how would you rate the participant’s overall engagement and participation during this session?” For *M&C-Y*: 1-Fair (Participant demonstrated reduced engagement; did not share thoughts, experiences, or goals without considerable prompting; and did not engage or attempt exercises.), 2-Good (Participant was engaged for the duration of the session; shared their thoughts, experiences, and goals with prompting; and participated in the exercise activities.), or 3-Excellent (Participant was engaged during the session and shared their thoughts, experiences, and goals in detail with minimal prompting and completed and engaged with exercises.). For *M&C-C*, the same 3-point rating structure was used, with the omission of the exercise component. An average engagement score was calculated for each participant (sum of scores/number of sessions attended) and at the group level (sum of participant scores/number of participants in the group). The *M&C-Y* and *M&C-C* groups were observed by 3 different research staff members. The primary study coordinator (AL) conducted 1-on-1 observer training on the participant engagement rating, incorporating didactic instruction and case review from the initial feasibility study [36,37]. Questions or uncertainties from alternate observers were discussed with the main study coordinator (AL).

##### Exit Satisfaction Survey

Youth and caregivers were invited to complete an exit satisfaction survey after the interventions were complete (T_1_). The survey asked about satisfaction with intervention components, the group setting, group size and timing, clinician facilitators, and future intent to implement strategies (5-point Likert scale; youth: 20 items; caregivers: 17 items). It also included 3 open-ended questions about the structure and delivery of the intervention, the best feature of the intervention, and what participants would like to see changed or improved.

##### Semistructured Interviews

Youth and caregivers had the opportunity to participate in an optional semistructured interview after the interventions were complete (T_1_) to better understand participants’ experiences with the interventions. All youth and caregivers who participated in the interventions were invited to participate in the interview through email. Toward the end of recruitment, the team focused on inviting families from underrepresented backgrounds, including fathers, boys, and participants from diverse ethnic backgrounds. Consistent with the concept of information power, sample adequacy was considered in relation to the study aims and the range of participant perspectives represented in the qualitative sample [67]. Interviews were completed virtually using Zoom Healthcare by a member of the research team who did not facilitate the intervention. Interviews were audio-recorded, transcribed verbatim, and checked for accuracy. Of note, the quotes presented in this paper were lightly edited to remove dysfluencies (eg, um) and repeated words for clarity and conciseness [68] and were labeled using data codes. See Multimedia Appendix 1 for the complete interview guides. Note that this interview guide was used in the broader pilot study and not all questions align with the scope of the research questions addressed in this manuscript. Additional qualitative findings will be presented in subsequent publications.

### Data Analysis

#### Overview

Results of the clinician delivery checklist, attendance tracking logs, and participant engagement ratings were reported using frequencies and descriptive statistics. Exit satisfaction survey Likert-scale question responses were collapsed into 3 groups: (1) favorable response (strongly agree, agree), (2) neutral response (neither agree nor disagree), and (3) unfavorable response (strongly disagree, disagree). They were then reported descriptively. Responses to open-ended exit satisfaction survey questions were grouped into initial codes by 2 members of the research team (AH, AL). Codes were then grouped into broader categories, and the frequency of responses in each category was reported. Two researchers (AH, AL) coded observer field notes descriptively, grouped related codes into categories, and presented categories related to engagement. Any discrepancies in the coding process were resolved through discussion. Findings from checklists, logs, field notes, surveys, and interviews were triangulated by assessing consistency and identifying complementary insights (eg, did observations align with participants’ reported experiences?) [57].

#### Interviews

Content analysis was used to examine the interview data, following both directed and inductive approaches. Pre-identified codes were derived from the multifaceted motivational framework of engagement [58]. Inductive coding was used to generate additional codes from the interview data to capture facilitators and barriers to engagement across both interventions. Facilitators were defined as factors that supported participants’ ability to engage, whereas barriers reflected factors that hindered their engagement. An initial codebook informed by the multifaceted motivational framework of engagement [58] and the concepts of facilitators and barriers to engagement was developed collaboratively by 2 coders (HAH, SMN) prior to coding. The 2 coders then independently applied the codebook to the interview transcripts and met regularly to discuss coding decisions, resolve discrepancies through consensus, and iteratively refine the codebook by clarifying existing codes and incorporating additional inductively derived codes. The analytic process involved (1) identifying topics or patterns, (2) gathering examples of topics from the transcripts, and (3) examining connections and relationships among codes to refine categories. This process included organizing examples of engagement using the predefined codebook and developing categories that reflected key facilitators and barriers. Quirkos software was used to apply codes and organize relevant excerpts [69].

#### Reflexivity

Interviews were conducted by AL (male research coordinator with a master’s degree in human health and nutritional sciences), HAH (female graduate student in clinical neuropsychology), and SMN (female research assistant with an undergraduate degree in neuroscience and psychology). Some interviewers had limited prior contact with participants through onboarding procedures. Interviewers did not conduct interviews with participants from a group if they acted as a clinician facilitator for that group. The broader study team has expertise in brain injury rehabilitation and experience with the *M&C-Y* and *M&C-C* interventions, which may have influenced the interpretation of the findings. To support reflexive practice and enhance trustworthiness, data analysis was completed by 2 individuals (HAH, SMN), and analytic memos documenting coding decisions and interpretations were maintained throughout the analytic process. Interpretations were also shared with the broader research team to encourage reflection and dialogue [70].

## Results

### Participants

**Figure 1.**
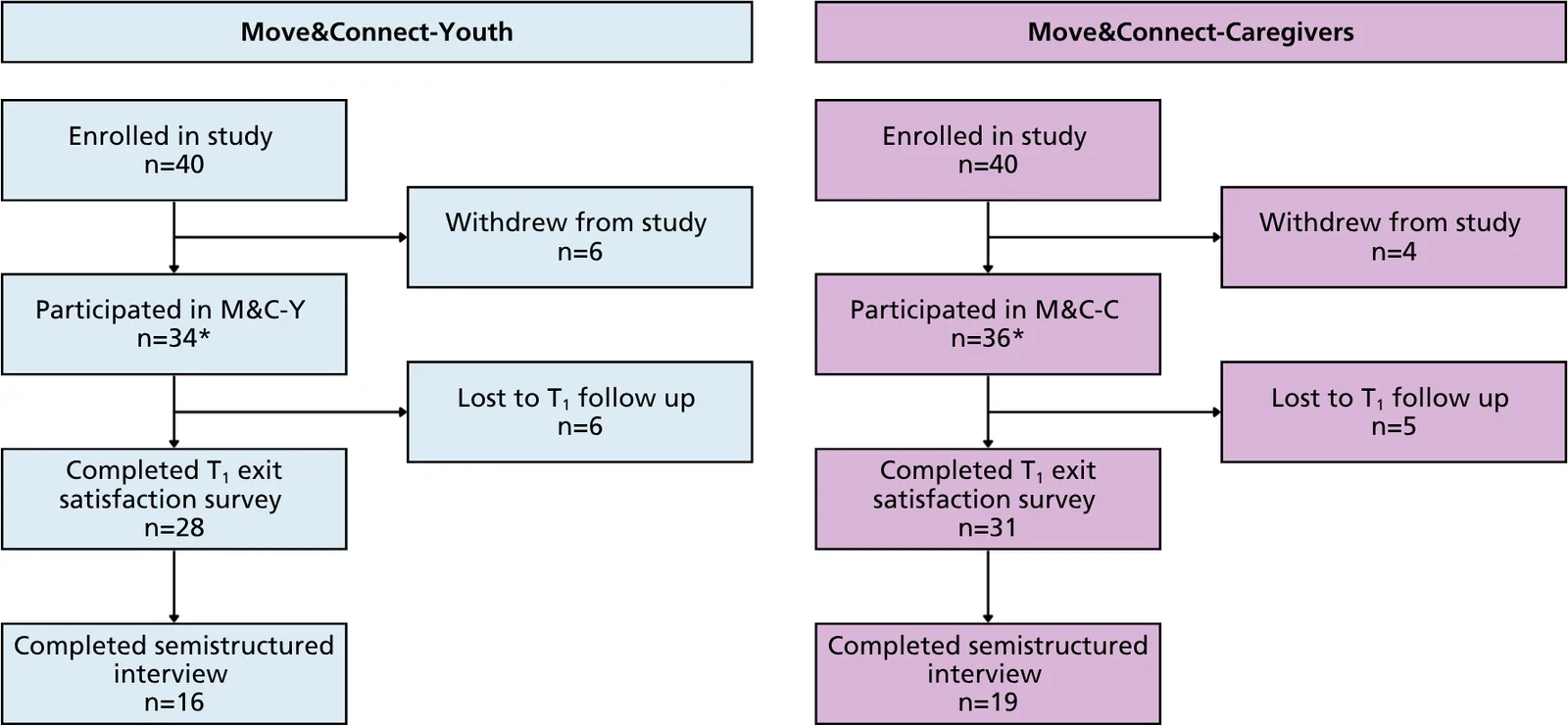
Youth and caregiver participant flow throughout the study, including participant numbers for enrollment, withdrawals, intervention participants, lost to T_1_ (immediately following invervention completion) follow-up, T_1_ exit satisfaction surveys, and semistructured interviews. *Demographics, attendance, and engagement ratings are reported for all participants who participated in the interventions.

Youth and caregiver participant progression throughout the study is outlined in Figure 1.

### Youth Participants

A total of 40 youth enrolled in the study (32 as dyads with parent/caregiver participants; 8 independents): 4 youth withdrew before the intervention began, 2 youth withdrew from the intervention due to scheduling demands, and 6 youth participants attended group sessions but were lost to T_1_ follow up.T_1_ follow-up procedures were completed by 28 youth. At the time of enrollment, youth participants completed a demographics form (see Table 2 for youth characteristics). All youth who attended T_1_ follow-up (n=28) were invited to an optional semistructured interview: 16 youth completed an interview (10 identified as girls, 5 identified as boys, and 1 identified as gender fluid), and 12 youth declined. All 6 *M&C-Y* sessions were attended by 7 of the 16 youth who were interviewed, and interviews lasted from 4 minutes to 27 minutes (mean 18 minutes). For clarity, this leaves the following sample sizes: demographics, attendance, and engagement ratings: n=34, 27 dyads, 7 independents; exit satisfaction survey data for fidelity of enactment: n=28, 21 dyads, 7 independents; and interviews: n=16, 11 dyads, 5 independents).

**Table 2.** Youth participant demographic information (n=34).

Demographic characteristics	Results
Age (years), mean (SD)	15.1 (2.28)
Age (years), range	9‐18
Sex, n (%)	
Female	24 (71)
Male	9 (27)
Prefer not to specify	1 (3)
Gender, n (%)	
Girl/woman	24 (71)
Boy/man	9 (27)
Gender fluid	1 (3)
Ethnicity, n (%)	
Black	3 (9)
Chinese	1 (3)
South Asian	3 (9)
West Asian	1 (3)
White	21 (62)
Multiple ethnic identities	5 (15)^a^
Health history, n (%)	
Learning disability^b^	9 (27)
Attention deficit hyperactivity disorder (ADHD)	8 (24)
Headache history prior to concussion	4 (12)
Anxiety diagnosis	8 (24)
Depression diagnosis	3 (9)
Other developmental disorders^b^	1 (3)
Other psychological/psychiatric conditions	4 (12)
Education history, n (%)	
Special education	5 (15)
Gifted program^b^	5 (15)
Individual education plan (IEP)	16 (47)
Previous concussion history^b^, n (%)	
0	9 (27)
1	11 (33)
2	3 (9)
3	5 (15)
4	3 (9)
≥5	2 (6)
Days since most recent concussion (at T_0_), mean (SD)	326.5 (380.97)
Days since most recent concussion (at T_0_), median (IQR)	198.5 (198.75)
Days since most recent concussion (at T_0_), range^c^	22‐1905
Setting/mechanism of injury, n (%)	
Sport	17 (50)
School	6 (18)
Motor vehicle collision	2 (6)
Fall	2 (6)
Assault	2 (6)
Accident (eg, from general clumsiness)	4 (12)
Other	1 (3)
Currently receiving therapy support for concussion, n (%)^b^	
Yes	18 (55)
No	15 (46)
Previous concussion support group attendance (in person or virtual), n (%)	
Yes	0 (0)
No	34 (100)
Current school involvement, n (%)	
Not going to school because of injury	3 (9)
Going to school, but in a less demanding way (eg, reduced hours/classes) compared to before injury	10 (29)
Going to school, just as I was before my injury	18 (53)
Other (eg, co-op, homeschool, graduated)	3 (9)
Job history, n (%)	
I am not working because of my injury	1 (3)
I am working, but in a less demanding way (eg, reduced hours/responsibility) compared to before my injury	1 (3)
I was not working before my injury, and I am not working now	26 (77)
I am working, just as I was before my injury	6 (18)

a White/Chinese, n=2; Greek/Arab/English/German, n=1; Asian/African, n=1; Japanese/Chinese/Trinidadian/White, n=1.

b 1 missing response (n=33).

c 1 participant was 22 days postinjury at T_0_ but >4 weeks postinjury at intervention onset.

### Caregiver Participants

A total of 40 caregivers enrolled in the study (32 as dyads with their child as youth participants; 8 independents): 3 caregivers withdrew before the intervention began, 1 caregiver withdrew during the intervention, and 5 caregivers attended group sessions but were lost to T_1_ follow-up. T_1_ follow-up procedures were completed by 31 caregivers. At the time of enrollment, caregiver participants completed a demographics form (see Table 3 for caregiver characteristics). All caregivers who attended T_1_ follow-up (n=31) were invited to an optional semistructured interview: 19 caregivers completed an interview (16 identified as women/mothers, and 3 identified as men/fathers), and 12 caregivers declined. All 6 *M&C-C* sessions were attended by 14 of the 19 caregivers who were interviewed, and interviews lasted between 13 minutes and 46 minutes (mean 26 minutes). For clarity, this leaves the following sample sizes: demographics, attendance, and engagement ratings: n=36, 27 dyads, 9 independents; exit survey data for fidelity of enactment: n=31, 21 dyads, 10 independents; and interviews: n=19, 11 dyads, 8 independents.

**Table 3.** Caregiver participant demographic information (n=36).

Demographic characteristics	Results
Age (years)^a^, mean (SD)	48.9 (5.92)
Age (years), range	35‐63
Sex, n (%)^b^	
Female	29 (83)
Male	6 (17)
Gender, n (%)^c^	
Girl/woman	28 (82)
Boy/man	6 (18)
Relationship to child, n (%)	
Mother	30 (83)
Father	6 (17)
Ethnicity, n (%)^b^	
Arab	2 (6)
Black	2 (6)
Chinese	1 (3)
Latin American	1 (3)
South Asian	1 (3)
Southeast Asian	1 (3)
West Asian	1 (3)
White	22 (63)
Multiple ethnic identities	4 (11)^d^
Education, n (%)	
High school	2 (6)
College	4 (11)
University	23 (64)
Graduate school	7 (19)
Household income (CAD), n (%)^c^	
<30,000	3 (9)
31,000‐50,000	1 (3)
51,000‐70,000	3 (9)
71,000‐90,000	2 (6)
91,000‐100,000	2 (6)
>100,000	23 (68)
Support group involvement, n (%)	
Currently attending any support group	2 (6)
Ever accessed a support group	11 (31)
Mental health/therapy support, n (%)	
Currently attending therapy/mental health support^b^	6 (17)
Ever accessed therapy support/mental health support	12 (33)

a 3 missing responses (n=33).

b 1 missing response (n=35).

c 2 missing responses (n=34).

d n=1 each: Chinese/Japanese, Metis/Ukrainian/Scottish, White/South Asian, Caribbean/German/Asian.

### Objective 1: Intervention Fidelity Assessment

This section describes the results of the intervention fidelity assessment for *M&C-Y* and *M&C-C*.

#### Fidelity of Design

An intervention mapping framework [35] was applied to the intervention design process for *M&C-Y* and *M&C-C*. Scratch and colleagues [36] and Al-Hakeem and colleagues [37] described the detailed intervention development process, alignment with theory, and feasibility testing results. The perspectives of youth, caregivers, and clinicians were incorporated throughout the development process. To ensure fidelity of the intervention design, an intervention manual was created that outlined the intervention purpose, format, and protocols required to complete *M&C-Y* and *M&C-C*. This was provided to clinician facilitators and research staff.

#### Fidelity of Training

Four occupational therapists and 2 physiotherapists were trained to deliver *M&C-Y*, and 2 social workers, 2 neuropsychologists, and 2 neuropsychology trainees were trained to deliver *M&C-C*. Our train-the-trainer model was underpinned by best practices and frameworks within the training field [71,72]. Train-the-trainer frameworks are commonly used to update health care professionals’ knowledge and skills and implement evidence-based interventions [71]. The training protocol used a blended learning approach, consisting of didactic and interactive methods, and was intended to ensure that all clinician facilitators delivered *M&C-Y* and *M&C-C* correctly, consistently, and safely to youth and caregivers. Feedback was also provided by the trainees (ie, individuals who were trained) about the training, which also informed the train-the-trainer approach. All clinician facilitators received institutional research training (eg, Tri-Council Policy Statement: Ethical Conduct for Research Involving Humans [TCPS-2], Collaborative Institutional Training Initiative [CITI]), orientation to the study protocols and procedures, and a study manual with all intervention materials (eg, slides, REDcap access) [66]. All clinician facilitators observed group sessions prior to facilitating the intervention independently and participated in 1-on-1, discipline-specific, peer debriefing throughout this process. Weekly debrief meetings were held between the clinician facilitators and the research staff observer to resolve any logistical or operational issues. Individual consultation with the study principal investigator occurred, as needed (eg, to review the mental health safety protocol).

#### Fidelity of Delivery

The *M&C-Y* intervention protocol was consistently delivered as intended across all 10 groups (discussion: 58/60, 97% sessions; exercise: 55/60, 92% sessions; goal setting: 57/60, 95% sessions). Reasons for modifying or deviating from the *M&C-Y* intervention protocol included sessions being canceled or modified due to only 1 participant attending (n=2), youth declining to participate in the exercises due to illness or being symptomatic (n=2), and discussions that were so productive that the exercise or goals setting components of the program needed to be shortened to accommodate session timing (n=2). The *M&C-C* intervention protocol was also consistently delivered as intended across all 9 groups (psychoeducation: 50/54, 93% sessions; activity: 50/54, 93% sessions; take home reflection [weeks 2‐6 only]: 37/45, 82% sessions). Reasons for modifying or deviating from the *M&C-C* intervention protocol included shortening the activity component because caregivers had questions and thoughts to share about the psychoeducation (n=3) or moving directly to the psychoeducation if caregivers did not complete the take home reflection (n=5). See Multimedia Appendix 2 for the detailed group breakdown of the clinician delivery checklist for *M&C-Y* and *M&C-C*.

#### Fidelity of Receipt

Attendance rates were as follows: all 6 sessions: 14/34 youth, 41% and 17/36 caregivers, 47%; 5 sessions: 8/34 youth, 24% and 7/36 caregivers, 19%; 4 sessions: 6/34 youth, 18% and 5/36 caregivers, 14%; 3 sessions: 4/34 youth, 12% and 4/36 caregivers, 11%; 2 sessions: 2/34 youth, 6% and 2/36 caregivers, 6%; 1 session: 1/36 caregivers, 3%. Participants frequently emailed the research team in advance when they were unable to attend a session (ie, due to scheduling conflicts or illness). In terms of fidelity receipt (ie, that participants understood and could use the intervention content), the research staff observer field notes revealed that participants frequently asked questions related to weekly topics (verbally and using the meeting chat function). Additionally, youth and caregivers were observed attending sessions with previously circulated educational materials and referring to them during group discussions. Participants completed weekly polls and discussions related to goal setting (youth) and take-home reflections (caregivers). Youth also frequently came to the sessions with the required supplies for the exercises (ie, running shoes, water bottles) that were provided in the weekly email reminders.

#### Fidelity of Enactment

For *M&C-Y*, the average exploratory participant engagement rating score was 2.72 (SD 0.36; range 1.5‐3.0), and the overall group engagement scores ranged from 2.37 to 3.0 (mean 2.73, SD 0.21; see Multimedia Appendix 2). Overall, youth participants were satisfied with the *M&C-Y* intervention, indicated by favorable responses across the exit satisfaction survey (Table 4). In addition, youth responses on open-ended questions in this survey revealed that youth perceived the best feature of *M&C-Y* to be connection with other participants (10/34, 36%), the weekly exercises (7/34, 25%), the weekly goal setting (6/34, 21%), and learning strategies that they could apply outside the sessions (2/34, 7%). In terms of changes or opportunities to improve *M&C-Y*, some youth recommended moving the intervention from a virtual to an in-person format (4/34, 14%), providing more opportunities for connection between participants (4/34, 14%), making the exercises easier or harder (4/34, 14%), and including different educational topics (4/34, 14%).

**Table 4.** Youth (n=28) and caregiver (n=31) exit satisfaction survey Likert-scale question results.

Questions	Frequency of favorable responses, n (%)	Frequency of neutral responses, n (%)	Frequency of unfavorable responses, n (%)
Youth exit satisfaction survey			
I felt that this program was a good use of my time	24 (86)	2 (7)	2 (7)
I felt that the topics covered were relevant to my personal experiences.	20 (71)	7 (25)	1 (4)
The exercise component of Move&Connect was beneficial for me.	20 (71)	1 (4)	7 (25)
The exercises were the right amount of difficulty for me.	16 (57)	5 (18)	7 (25)
The exercise component was an appropriate length of time.	20 (71)	6 (21)	2 (7)
The educational component of the Move&Connect program was beneficial for me.	23 (82)	3 (11)	2 (7)
The program content (slides/handouts) complimented the discussions.	24 (86)	4 (14)	0 (0)
The discussions lasted an appropriate amount of time.	24 (86)	2 (7)	2 (7)
The goal setting component of the Move&Connect program was helpful for me.^a^	22 (81)	4 (15)	1 (4)
I will start/continue to set goals related to my recovery.	20 (71)	4 (14)	4 (14)
The group size was appropriate.	22 (78)	3 (11)	3 (11)
The sessions lasted an appropriate amount of time.	23 (82)	2 (7)	3 (11)
Meeting other youth with similar experiences was important for me.	23 (82)	4 (14)	1 (4)
I was comfortable sharing my experiences with other youth in a group setting.	22 (78)	3 (11)	3 (11)
I was comfortable completing the exercise portion of the program in a group setting.	23 (82)	4 (14)	1 (4)
I would recommend this program to other young people or friends who have had a concussion.	23 (82)	3 (11)	2 (7)
I felt supported by the group facilitators.	27 (96)	1 (4)	0 (0)
I felt supported by other members of the group.	23 (82)	3 (11)	2 (7)
I felt that the other members of the group understood my experiences.	19 (67)	8 (29)	1 (4)
I felt connected to the other members of the group.	15 (54)	7 (25)	6 (21)
Caregiver exit satisfaction survey			
I felt that this program was a good use of my time.	31 (100)	0 (0)	0 (0)
I felt that the topics covered were relevant to my personal experiences	31 (100)	0 (0)	0 (0)
I felt that the take-home activities were beneficial to my experience with the program.	23 (74)	6 (20)	2 (6)
The program handouts/resources were relevant and supplemented the program sessions well.	23 (74)	7 (23)	1 (3)
The educational component of the Move&Connect program was beneficial.^b^	29 (97)	1 (3)	0 (0)
The activities/strategies taught in the program were applicable.	28 (90)	3 (10)	0 (0)
I will start/continue to apply the activities/strategies taught during the program.	27 (87)	4 (13)	0 (0)
I found the weekly discussions to be helpful.	30 (97)	1 (3)	0 (0)
The group size was appropriate.	27 (87)	2 (6)	2 (6)
The sessions lasted an appropriate amount of time.^b^	28 (93)	2 (7)	0 (0)
Meeting other parents with similar experiences was important for me.	30 (97)	1 (3)	0 (0)
I was comfortable sharing my experiences in a group setting.	31 (100)	0 (0)	0 (0)
I would recommend this program to other caregivers or friends.	29 (94)	2 (6)	0 (0)
I felt supported by the group facilitators.	31 (100)	0 (0)	0 (0)
I felt supported by other members of the group	29 (94)	2 (6)	0 (0)
I felt that the other members of the group understood my experiences.	30 (97)	1 (3)	0 (0)
I felt connected to the other members of the group.	26 (84)	4 (13)	1 (3)

a 1 missing youth response (n=27).

b 1 missing caregiver response (n=30).

For *M&C-C*, the average exploratory participant engagement rating score was 2.79 (SD 0.27; range 2.0‐3.0), and the overall group engagement score ranged from 2.5 to 3.0 (mean 2.8, SD 0.17; see Multimedia Appendix 2). Caregiver participants were also satisfied with the *M&C-C* intervention, with favorable responses indicated across the exit satisfaction survey (Table 4). Through open-ended questions on this survey, caregiver participants also indicated that they enjoyed the convenience of the virtual format (7/31, 23%) and that the best features of *M&C-C* were connecting with other caregivers with similar experiences (19/31, 61%) and the education and strategies provided (11/31, 36%). Suggestions to improve *M&C-C* included providing different educational topics and resources (6/31, 16%), additional reminder emails with program content (5/31, 16%), allowing more opportunities for connection between participants (4/31, 13%), modifying the timing of sessions (3/31, 10%), and increasing the number of participants in each group (2/31, 7%).

Examples of how youth and caregivers (Table 5) engaged with the interventions are outlined using the motivational framework from King et al [58], followed by an examination of the individual, intervention-related, and contextual factors that influenced these experiences of engagement (Objective 2). Behavioral engagement in both interventions centered around taking part in intervention components (eg, discussions and group activities) and participants’ confidence in their ability to perform intervention tasks. Affective engagement involved the positive feelings and attitudes participants had toward the interventions. Cognitive engagement included participants’ interests, ways they felt inspired, and an appreciation of the structure and utility of *M&C-Y* and *M&C-C*.

**Table 5.** How youth and caregivers engaged with *Move&Connect-Youth* (*M&C-Y*) and *Move&Connect-Caregivers* (*M&C-C*), with each example of behavioral, affective, and cognitive engagement presented with an illustrative participant quote.

Examples	Quotes
*M&C-Y*	
Behavioral (participation/confidence in ability to do intervention tasks)	
Group discussions	We would talk about topics, and it would be nice to hear people’s opinions and ideas on how to help […] it helped sometimes, it is not bad at all (Y01, age 11 years).
Goal setting	I was sharing my goals with everyone and they were also, they also had goals for themselves, kind of was a motivation to continue out for that, that goal. And... so I can come back next week and say that I accomplished it (Y09, age 17 years).
Exercising	I thought it was really cool to do all the exercises and have good discussions (Y10, age 16 years).
Beliefs about self-efficacy	I think I’ll be able to. Uh, if you know a goal comes up that I think I will be able to follow it and accomplish it pretty well (Y16, age 17 years).
Affective (emotional investment and attitude towards intervention)	
Comfort and keenness	It made me feel like if I can do it, they can do it. If they can do it, I can (Y01, age 11 years).
Active and energized	I think it made me feel less tired, uh, just like yeah just less tired and more felt better, yeah, just like more active (Y11, age 16 years).
Cognitive (conviction of intervention appropriateness)	
Inspired by peers	It was inspirational to see them [the other participants] getting better throughout the weeks (Y14, age 17 years).
Belief in utility and structure	It was really educational, I think, and it was really good to talk to other people that were like experiencing the same thing as me. I think that was the most beneficial part for me (Y12, age 16 years).
*M&C-C*	
Behavioral (participation/confidence in ability to do intervention tasks)	
Emotionally vulnerable discussions	We were all a part of a group that was sharing information and relating to each other (C17).
Active listening and verbal participation	When I would make a comment and the other parent would respond by saying, “yeah, I get that, I get that I have that too.” And then I, you know, the reciprocal was there (C02).
Application of strategies outside of sessions	It brought a heightened awareness about that might be what the children have been going through, right, and, and just being more aware and understanding about everything that is going on (C12).
Affective (emotional investment and attitude towards intervention)	
Validation and connection	It was great connection and kind of, it’s nice to be able to talk about it with people who are going through similar experiences (C03).
Relief	I don’t know how else to say it but it was it really was umm liberating. I don’t know if that’s the right word, but I just felt like a huge relief of saying, okay, I’m not the only one. I’m not going crazy when my kid is actually experiencing this, and you know somebody else actually finally understands me (C04).
Cognitive (conviction of intervention appropriateness)	
Relevance and benefit of *M&C-C*	I’m actually learning something, the presentation is good, you know? This is knowledge coming to me that I didn’t have before, so yes (C11).
Positive perceptions of *M&C-C* structure	I thought it was just, really well run. It was just enough of everything like do you know what I mean like, um, education versus, um, chatting, um, yeah, I just thought it was done really well (C12).

### Objective 2: Facilitators and Barriers to Engagement

**Figure 2.**
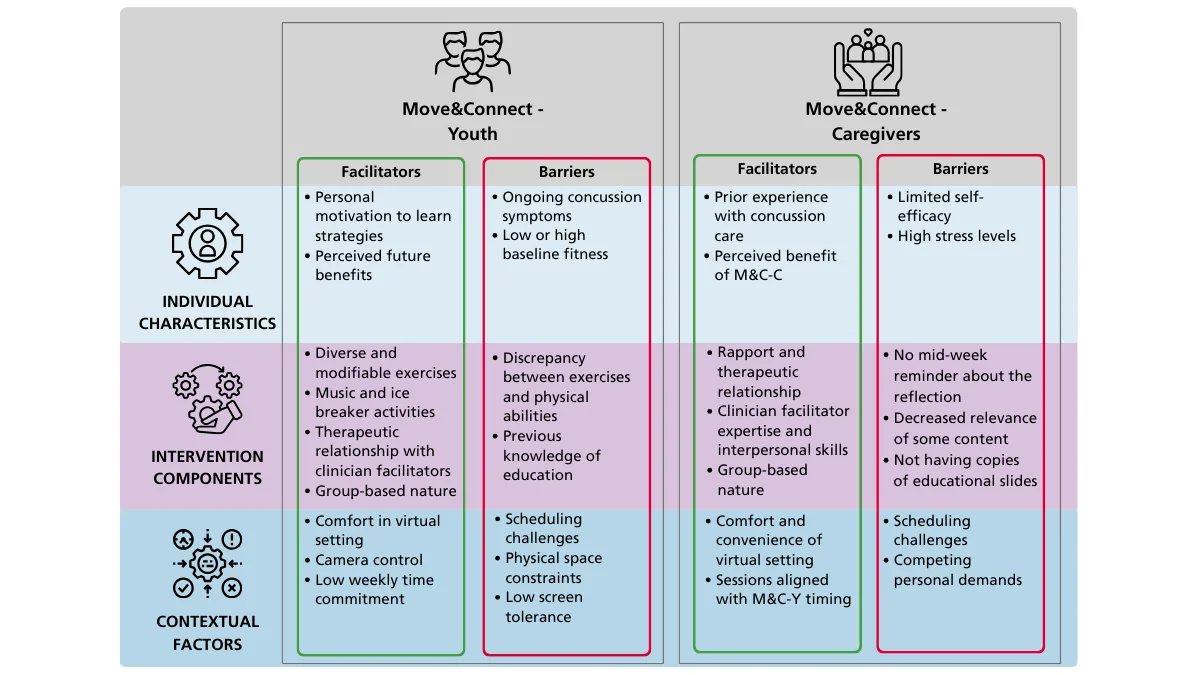
Visual representation of key barriers and facilitators to engagement in *Move&Connect-Youth* (*M&C-Y*) and *Move&Connect-Caregivers* (*M&C-C*), organized by individual characteristics, intervention components, and contextual factors.

This section describes factors that influenced participant engagement within *M&C-Y* and *M&C-C*. Facilitators and barriers to intervention engagement are organized across 3 overarching categories: (1) individual characteristics, (2) intervention-related components, and (3) contextual considerations (Figure 2). Findings for *M&C-Y* are presented first, followed by those for *M&C-C*.

#### Facilitators of Engagement in M&C-Y

##### Individual Characteristics

Individual motivation and personal interests facilitated engagement. With respect to motivation, youth described joining *M&C-Y* to improve health, manage symptoms, and return to valued activities. Some also noted an interest in contributing to research and helping others.

##### Intervention Components

A lighthearted, comfortable, and nonjudgmental environment was reported to drive engagement in *M&C-Y*. Youth described *M&C-Y* sessions as friendly and positive, with a lighthearted tone that included laughter and playful moments among participants and clinician facilitators:

We have like those like ball throwing or sock throwing things, and things would just fly everywhere. [We would] like poke fun at each other but like in a positive way that made you feel like you can like laugh at yourself and not feel so bad if you’re not good at something.

[Y15, age 17 years]

Creative icebreaker activities and conversation prompts were reported to ease nerves and encourage engagement. Youth noted that the personalized music played in the session supported the active rehabilitation exercises. Youth also emphasized that seeing their peers being open and transparent regarding their symptoms and challenges made it easier to participate in discussions and relate the intervention content to their own situations:

They [the other participants] were really open to sharing, which I really appreciated. And they shared some similar things. And so it was kind of like, I can share something like this. I felt comfortable sharing, some of the stuff that I was dealing with, because they were also sharing some of the stuff they were dealing with and it was similar.

[Y12, age 16 years]

Clinician facilitators were consistently described as supporting youth engagement. Youth commented that the clinician facilitators’ style felt relatable, and they offered encouragement and validation. Clear reminders about confidentiality or “what’s said on the call stays on the call*”* (Y01, age 11 years) reinforced trust and made disclosure feel appropriate within the group. Further, youth emphasized that the clinician facilitators taking part in activities and exercises themselves, alongside participants, enhanced engagement and comfort.

Most youth described the educational content as helpful and relatable when it linked concussion information to their own symptoms, daily routines, and recovery goals. They identified the variety of exercises and flexibility as supportive and appreciated that the exercises were tailored to different abilities and broken down into manageable blocks:

There were a lot of options, so even if I did not want to do one, if I was doing an exercise for a long time and could not do it anymore, I could move to an easier version.

[Y15, age 17 years]

Youth also valued that the exercises were challenging enough to feel meaningful without being overwhelming:

They [the exercises] were not too hard where I would be sore the next day, but they were hard enough that I would be tired in the end. That helped my physical and mental health, and they were fun to do.

[Y11, age 16 years]

Youth noted that watching peers share openly encouraged their own participation and improved feelings of connection. More specifically, having others in the group of similar age with comparable concussion experiences was helpful:

Everyone was always smiling, um, like there was never any criticism or anything, any questions were answered like there was kind of always a solution, like yeah it was just a nice environment. And again, with the age like being so close and similar in age and experiences too that it just it was a comfortable environment.

[Y16, age 17 years]

Importantly, the group format supported accountability and follow-through with setting and achieving goals. Youth indicated that the clear expectation to set goals, return the following week, and report on progress to the group promoted accountability. Others described how hearing peers’ goals provided ideas for their own targets and a sense of shared effort:

I did set goals [before M&C-Y], and I tried to stick to them, but I just, I found I really wasn't sticking to them at all. And then within the group, it really helped me to stick to them because […] I was sharing my goals with everyone and they were also, they also had goals for themselves, kind of was a motivation to continue […] so I can come back next week and say that I accomplished it.

[Y09, age 17 years]

A small group size was also a facilitator to engagement. Youth described that a smaller group made it easier to speak, reduced stress levels, and increased the sense that individual contributions would be heard. It also allowed for more direct check-ins and time from the clinician facilitators supporting the sessions:

There weren’t many people, and I felt like my voice was heard a lot of the time.

[Y04, age 16 years]

##### Contextual Factors

Broader environmental and technological factors also facilitated *M&C-Y* engagement. Youth were able to attend sessions more regularly when they had fewer extracurricular and school-related commitments. The virtual format supported comfort and engagement for some youth, particularly in their ability to control the camera and position themselves at a comfortable distance from the camera:

You don't actually have to see people like super close up. You can kind of move away from your camera and just focus on yourself instead of, oh, what are other people doing? Or what are other people thinking about watching me exercise.

[Y06, age 16 years]

### Barriers to Engagement in M&C-Y

#### Individual Characteristics

Ongoing concussion symptoms (eg, headaches, fatigue, dizziness) were barriers to full participation in the exercises or made it harder to attend sessions. One youth described that where they were in their concussion recovery affected their participation and did not feel that the exercise component aligned with their needs:

I don’t think exercises are really on par with my recovery. Because I’ve noticed that exercise is actually in fact making my symptoms worse. Even if it’s just light exercises like walking. So, I wouldn’t say that it was bad. Once again, I would say it just wasn’t for me per se.

[Y05, age 17 years]

#### Intervention Components

Having exercises that felt too easy or too difficult was a barrier to engage fully in the session, particularly when youths perceived a mismatch between their current abilities and the session of intensity. For example, 1 youth who competed in track and field wanted “more challenging exercises,” noting that even the harder options felt easy at times (Y08, age 15 years). However, the availability of modifications often mitigated some of these barriers:

I liked how they had very different variations depending on whether you could do it or not. I cannot really jump a lot because I get dizzy, and they have different alterations to some of the exercises.

[Y06, age 16 years]

Several youth reported that portions of the education content did not feel new nor relevant to their stage of recovery and were described as repetitive. For some, prior exposure to concussion resources or longer recoveries meant they had previously received education, which impacted how they engaged with the material. Others noted that, although the education was acceptable, it did not provide additional ideas for self-management beyond what the participant was already doing:

If people who are starting to realize what [concussion] is and what to do. And to me, I feel like I was a little bit of a step ahead. So I had to learn all this stuff already by myself, but it was still a good kind of process.

[Y03, age 15 years]

Youth offered suggestions to improve relevance and depth of education materials, including expanding topics such as sleep hygiene (Y01, age 11 years), brain anatomy, and nutrition (Y05, age 17 years). They also suggested integrating interactive elements or games into the education to increase engagement with younger youth. Some requested additional time for open-ended and peer-to-peer discussion to share experiences more fully:

I do wish I had more time to kind of have like more open-ended discussions like between like everyone. Just a bit more like time like specifically for having the discussions between all the kids. And like how they feel and their experiences…

[Y15, age 17 years]

A barrier to group participation involved social comparison, with 1 participant commenting that they evaluated themselves against others in terms of their recovery:

It was kind of like, ughh, you know, ‘wow I’m so much worse than everyone else here.’ It was kind of like a little not nice but yeah, whatever, like again it doesn’t really affect me whether they’re better than I or not as good, so yeah.

[Y16, age 17 years]

#### Contextual Factors

Scheduling demands, physical space, and screen tolerance were noted as barriers to engagement. Scheduling commitments related to schoolwork and extracurricular activities impacted attendance. One youth also commented that physical space in their home was a barrier to participating in the exercises:

I was doing it in my room, and I'm tall, my ceiling’s a bit low, so it was a bit difficult at times.

[Y04, age 16 years]

Some youth noted limited screen tolerance made paying attention harder during the session. One youth, however, noted gradual improvement in screen tolerance over the course of the intervention:

At first, I was like ‘how can I get on a Zoom call because screens hurt my eyes?’ [yeah] but I tried it the first time. I wasn’t feeling well that first week, but, I felt better as we got into the exercises. And then I also wasn’t as afraid of going on to screens anymore, so yeah.

[Y14, age 17 years]

### Facilitators of Engagement in M&C-C

#### Individual Characteristics

Individual characteristics that facilitated caregivers’ engagement in *M&C-C* were motivation, perceived benefit of the intervention, and prior experiences with concussion care. Participants were motivated to join *M&C-C* to gain practical strategies to support their child’s recovery, enhance their own well-being, and increase their understanding of concussion. Many caregivers were also interested in connecting with others in similar situations. Their frustration with previous concussion care also motivated them to join *M&C-C*:

I think we were at our wit’s end right? And […] no matter where we were going, the doctors… we, we just felt like we weren't being heard, right?

[C04]

#### Intervention Components

Elements of the intervention that promoted engagement in *M&C-C* were having a dedicated space to connect and receive validation from other group members; clinician facilitator expertise; and a small, cohesive group format. Caregivers shared that the group provided a unique opportunity for open discussion with others who understood the challenges of supporting a child through prolonged concussion recovery, as a caregiver stated “No one judged me here” (C17). Connecting with other caregivers, exchanging stories, and hearing about others’ coping strategies were among the most helpful aspects of the sessions. Similarities among members also supported engagement, with caregivers highlighting that their group happened to include parents in similar caregiving roles with similar aged children as them, which appeared to ease sharing among one another:

Just recognizing things that I was like oh yes I, I’ve got that too, and, and so, you know, recognizing the commonalities that are there and kind of then kind of what they’re going through and how they deal with it was really nice.

[C18]

Caregivers reported feeling listened to and encouraged to participate, noting that the facilitators maintained a steady pace, balanced education with support, and created space for discussion. Sessions were described as well-structured and smoothly run, with facilitators who were personable, responsive to questions, experts in their fields, and clear in presenting material:

[The clinician facilitators] were just phenomenal. Besides the fact of being very personable, I think just the ability to ask some questions and the way they took us along the program. You know it, they were just really well-handled, well-structured sessions.

[C04]

Small group size was consistently mentioned as an important facilitator of engagement in *M&C-C*. Participants reported that fewer members increased connection, made communication easier, and fostered a sense of responsibility to attend and contribute. One caregiver shared:

This was the smallest group I've been involved with, and I think that the benefits of these small numbers really… they can't be ignored. […] I found, like I said, the level of intimacy was very beneficial and not only did it drive engagement, it really helped me make a full commitment to the program.

[C02]

#### Contextual Factors

Caregiver engagement was also facilitated by environmental and technological considerations. Caregivers highlighted that it was helpful to have both sessions (*M&C-Y* and *M&C-C*) run at the same time, as their children were occupied and not listening in on the caregiver group. The virtual format was widely viewed as convenient by caregivers. They emphasized that the virtual format reduced travel time and fit more easily into busy schedules. The ability to join from home was described as feasible and manageable:

I wouldn’t have been able to do those [the sessions] if it wouldn’t have been over Zoom because being so far away there, there’s no way I would have been able to go for an hour and spend 3 hours getting there and back, wouldn’t have made it work.

[C17]

### Barriers to Engagement in M&C-C

#### Individual Characteristics

Some caregivers had initial doubts about their ability to understand the intervention content:

You’re thinking, oh god, you know, is this going to be, uh, a really science-y thing, and I don’t understand science.

[C19]

Others noted that stressful life demands made it challenging to fully engage in sessions or implement strategies between sessions:

It’s still been hard to implement some of this only because I’m being pulled in so many different directions.

[C04]

#### Intervention Components

Although information shared was generally viewed positively, caregivers identified practical barriers in how materials were delivered by facilitators. Some requested receiving the full slide deck in advance to review and save for later use, as well as accessibility improvements such as larger font to make slides easier to read. Several caregivers also suggested sending additional reminders to complete the optional reflection activities:

I was remiss at not doing my first week’s homework. And the reason why is because I couldn't remember what it was. And you know, had too many distractions on…

[C06]

Caregivers acknowledged that a limitation of the group model is that not all content resonated equally with all participants, with some material feeling less applicable to their circumstances:

Some weeks are maybe like a little bit longer or more dull if you want to say but that’s always the way, right. You're going to latch on to like, some of the other parents latched on to things that I was like, I don't see myself or my kid in any of this. And then, so those weeks seem a little bit less connecting but that is the nature of different people being different.

[C07]

#### Contextual Factors

Time and logical constraints were common barriers to engagement. Caregivers described feeling stressed and overwhelmed with other life demands, which made it difficult to attend consistently, complete between-session activities, or implement new strategies. Scheduling the sessions during the evening (ie, 5 PM or 6 PM) was described as challenging due to appointments and family routines. Caregivers proposed alternative scheduling to improve engagement, such as later evenings, weekend options, or condensed workshop formats to broaden access. Some suggested that weekend sessions might attract caregivers who were underrepresented in the groups:

So, if it was a weekend workshop, maybe you'd get some of those dads out. Maybe they could go for a weekend. I could see them being like, okay, let’s get those done in a one-er, you know?

[C06]

Despite the virtual convenience, a few caregivers also expressed a preference for at least one in-person touchpoint, such as a closing session or celebration, to strengthen personal connections.

## Discussion

### Principal Findings

Within the context of a broader pilot study, we used a multimethod approach to assess the fidelity of the *M&C-Y* and *M&C-C* interventions in terms of intervention development, training, delivery, receipt, and enactment. Second, we explored barriers and facilitators to youth and caregiver engagement with the interventions at the individual, intervention, and contextual levels. A rigorous approach to fidelity monitoring ensured high adherence to our theory-driven intervention protocols, quality of virtual delivery, and participant satisfaction and engagement. Caregivers showed consistent and high levels of satisfaction with *M&C-C*, whereas youth satisfaction with *M&C-Y* had some variability, particularly related to exercise difficulty. Both youth and caregivers expressed that clinician facilitators established a supportive, nonjudgmental environment that was central to building feelings of comfort and supported engagement. The group format provided important validation of youth and caregiver experiences and supported accountability to apply strategies outside of the interventions. Barriers to youth engagement included concussion symptoms, baseline fitness, and previous knowledge of education, whereas barriers to caregiver engagement included stress, scheduling demands, and limited access to intervention materials. Results provided important implications for delivering virtual group interventions for youth with PSaC and their caregivers.

### Comparison With Prior Work

Our approach to fidelity assessment aligned with the framework from Bellg and colleagues [55] and best practices for evaluating intervention fidelity [53,54] by using multiple methods to allow for triangulation and reducing the chance of biases associated with any one approach [73]. Triangulation recognizes that each individual will have a unique perspective on a shared phenomenon [57]. We used clinician facilitator checklists and research staff observer logs and field notes and provided multiple mediums for youth and caregivers to provide their feedback. There is heterogeneity in what constitutes “good” attendance of an intervention [74]; however, it is promising that the majority of participants in this study attended at least 5 of 6 sessions. As recommended by Toomey and colleagues [75], our approach extended beyond intervention delivery to include an evaluation of fidelity receipt and enactment. Opportunities to assess fidelity receipt and enactment were also built directly into the interventions to reduce administrative burden [57]. A rigorous approach to fidelity assessment is especially important in complex interventions like *M&C-Y* and *M&C-C* that likely have multiple potential active components, and this fidelity evaluation can enhance replicability and help ensure that future outcomes can more confidently be attributed to the interventions [53,57,75].

Virtual videoconferencing technology (eg, Zoom Healthcare) enables access and scalability of pediatric rehabilitation interventions [76,77], and families report that virtual care offers benefits such as convenience, flexibility, and comfort in the home environment [78]. Similar to findings reported by Shore and colleagues [26], where youth with PSaC and parents appreciated the convenience and comfort of engaging in individual virtual rehabilitation from home, participants in this study described the virtual setting as a facilitator of attendance and felt comfortable completing the interventions from home. Although participants appreciated the benefits provided by the virtual setting and almost all participants felt supported by the other members of the group, some felt that they did not connect with other members of the group. The discontinuous nature of cyberspace or the “loss of shared space” in virtual therapy has been identified as a threat to group engagement and relational processes in virtual groups [79,80]. To increase connection and engagement, some participants requested an in-person component or more time for unstructured or open-ended conversations between participants. The virtual nature of *M&C-Y* and *M&C-C* may lack the informal opportunities for connection that happen in the waiting room or parking lot before and after the weekly sessions. Indeed, there seems to be a trade-off between convenience and connection in the virtual group setting for youth with PSaC and caregivers.

The findings from this study have important implications for providing virtual group-based education and active rehabilitation to youth with PSaC. Active rehabilitation and education are recommended in the treatment of PSaC [12], and virtual active rehabilitation interventions are emerging as feasible approach to care [22,23]. The educational and exercise components of *M&C-Y* were delivered as intended in the majority of sessions, which is especially important as intervention personalization and deviations have been poorly reported in previous exercise interventions for concussion [81]. Although most youth agreed the education was beneficial and relevant to their personal experiences, some youth described that the education was repetitive, with information that they had already received from other health care providers or strategies that they had to previously learn on their own. Importantly, youth in this study had many risk factors for prolonged recovery [2,82] and were an average of almost 1 year postinjury (mean 327 days). As such, perspectives of these youth and their caregivers, as well as their responses to the interventions, could be different than youth injured more recently. Consistent with current clinical care guidelines [12], this suggests that education should be provided earlier in the recovery process.

The *M&C-Y* program aligns with a recent call-to-action to develop concussion interventions that do not require expensive gym-based equipment (eg, stationary bike) and to implement home exercise programs via virtual care that only require accessible and affordable materials [83]. Interestingly, both high and low levels of baseline fitness were identified as a barrier to engagement in the exercise portion of *M&C-Y*. The intervention uses a predetermined set of exercises, and clinician facilitators provided modifications for each exercise to make it easier or more challenging, which was viewed as a facilitator to engagement for participants. Even with these modifications, some youth felt that the exercise component was not the right level of difficulty. This is consistent with previously identified challenges with personalizing exercise in the virtual group setting, including decreased direct observation [84] and not being able to use your hands to correct exercise techniques [85]. Future delivery of *M&C-Y* could mitigate this by providing an individual assessment before the program begins or further individualize the exercises by providing additional opportunities for youth to provide feedback on the exercises throughout the intervention. This aligns with the principles of client and family-centered care, which emphasizes the importance of working with youth with PSaC to align aspects of the intervention to their needs [44].

There are limited educational interventions designed specifically for caregivers of youth with PSaC [34]. Unlike youth in this study who described receiving education about managing their concussion from multiple health care providers, *M&C-C* was the first time that the caregivers received education designed specifically for them. Caregivers were overwhelmingly satisfied with *M&C-C,* and virtually all caregivers agreed that the facilitators were supportive, meeting other caregivers with similar experiences was important, and the discussions were helpful and relevant to their personal experiences. These findings reinforce the importance of developing and evaluating caregiver-centered interventions and support [34]. Caregivers of youth with PSaC report experiencing high levels of stress [28] and being overwhelmed managing multiple health care appointments to support their child [44]. Similarly, caregivers in this study also identified high levels of stress and busy conflicting schedules as barriers to attendance and engagement in *M&C-C*. This was apparent, as the most common modification to the *M&C-C* intervention protocol was omitting the take-home reflection review due to caregivers not completing the reflection between sessions. Strategies like customizing mid-week reminders about reflections were suggested to support caregivers to more fully engage in the intervention.

### Limitations

This study has limitations worth noting. First, as is the case with most intervention studies, results from the T_1_ satisfaction surveys and interviews only include the individuals that attended the T_1_ data collection session and may represent the perspectives of participants who were most engaged with the interventions. To ensure a balanced approach to intervention receipt and enactment, data from individuals who were lost to T_1_ follow-up were included in the demographics, attendance, field notes, and engagement ratings. This is important, as individuals with concussion who do not attend follow-up are vastly underrepresented in the literature [86]. As participation in the interviews was optional, the qualitative findings may also be subject to self-selection and positivity bias and may not fully represent the experiences of all intervention participants. There is also potential of nonindependence of observations from members of the same family. In addition, fidelity receipt and enactment were assessed primarily through indirect observer field notes, engagement ratings, and self-report. The participant engagement rating was developed by the research team, as we were unable to identify a validated, observer-rated measure of in-session engagement that reflected the components of the interventions and was feasible for the virtual group context. Although the scale provided useful exploratory insights, its reliability and validity have not been established and therefore may not capture the intended construct of engagement. The scale may also be subject to observer bias, as sessions were not recorded and observed by a single rater. To mitigate this, our engagement evaluation was multifaceted, and triangulation occurred with surveys and interviews. Additionally, recruitment for this study primarily took place in a hospital-based clinic in a large urban center where individuals may have had increased access to concussion care in general. Although the virtual format of *M&C-Y* and *M&C-C* makes the interventions more accessible to individuals living in rural areas, future efforts should be made to provide access to the interventions and evaluate it in this group. In addition, most participants in this study identified as White and were from high socioeconomic status households. There is a dearth of information about the association between socioeconomic status and concussion; however, recent Canadian studies have identified that individuals from households with lower socioeconomic status are less likely to access care for concussion [41,87,88]. Understanding how individuals from diverse backgrounds engage with the *Move&Connect* program should be a priority for future research. Strategies to engage these underrepresented groups could include providing alternative scheduling, offering translation services, and a focus on community-based recruitment. Last, most participants in this study identified as girls/women. This is consistent with findings from initial feasibility testing of *M&C-Y* [36] and *M&C-C* [37] and may indicate a preference by girls/women toward group-based interventions. Future efforts should be made to understand the experiences of boys/men with the interventions and explore barriers they may experience when enrolling in this type of intervention.

### Conclusions

This paper aimed to evaluate the intervention fidelity of *M&C-Y* and *M&C-C* and explore facilitators and barriers to youth and caregiver engagement with the virtual group interventions. The theory-driven fidelity evaluation, including intervention development, training, delivery, receipt, and enactment, demonstrated that the pilot study of *M&C-Y* and *M&C-C* was administered with high delivery adherence. Caregivers reported high levels of satisfaction and engagement, whereas youth satisfaction and engagement had some variability, particularly related to the exercise component. Engagement with the interventions was shaped by individual, intervention, and contextual factors. Important considerations for facilitating virtual group interventions for youth with PSaC and their caregivers are presented. Future research should examine the impact of *M&C-Y* and *M&C-C* on outcomes such as concussion symptoms, goal attainment, self-efficacy, mental health, and family functioning.

## Supplementary material

10.2196/88771 Multimedia Appendix 1 Youth and caregiver interview guides.

10.2196/88771 Multimedia Appendix 2 Clinician delivery checklist results and group engagement scores.

10.2196/88771 Checklist 1 COREQ (Consolidated Criteria for Reporting Qualitative Studies) checklist.

## Abbreviations

CITI: Collaborative Institutional Training Initiative
COREQ: Consolidated Criteria for Reporting Qualitative Studies
FAB: family-directed approach to brain injury
HBKRH: Holland Bloorview Kids Rehabilitation Hospital
*M&C-C*: Move&Connect-Caregivers
*M&C-Y*: Move&Connect-Youth
PSaC: persisting symptoms after concussion
TCPS-2: Tri-Council Policy Statement: Ethical Conduct for Research Involving Humans
