# Supplementary material for: Delivering Virtual Group Interventions for Youth With Persisting Symptoms After Concussion (Move&Connect-Youth) and Their Caregivers (Move&Connect-Caregivers): Fidelity and Engagement Evaluation
Source: JMIR Rehabil Assist Technol. 2026 Sep 21;13:e88771. doi: 10.2196/88771 (PMC13592660; doi:10.2196/88771)
Supplement: Supplementary file 2 [file rehab-v13-e88771-s001.docx]

**Youth Interview Guide**

Through this interview, I hope to learn about your experience with the Move&Connect-Youth program. The questions will be open-ended to allow you the chance to raise the issues that you feel are important. You do not have to answer any question you feel uncomfortable with, and you can stop the interview at any time. You can share as much or as little as you like. Your responses will be confidential and anonymized in any findings we may publish. Quotes from the interviews may be used, but it will not be possible to identify their source. Your responses will not affect the care your child receives at Holland Bloorview Kids Rehabilitation Hospital.

Please note the interview will be audio recorded so that the research team can keep a record of the interaction.. The recording will be deleted following transcription and won’t be used for any purpose other than the ones outlined. This record will let the team review and analyze the interview responses along with other participants’ feedback to help with our research study. And, I will be happy to answer any questions. Do you have any questions for me before we begin?

**General Questions**

1. Can you tell me what it was like to participate in *M&C-Y*?

- Can you expand on what you did/didn’t like about that aspect of the program?

2. What have made you decide to join *M&C-Y*?

- What did you hope to get out of *M&C-Y*?
- Did you have any reservations about joining *M&C-Y*? If so, how were they overcome?

3. What made you come back to *M&C-Y* each week?

- Was there anything in particular that supported/encouraged you to stay engaged with *M&C-Y*?

**Goal setting**

4. As you know, part of the group included setting goals each week. Prior to *M&C-Y*, did you have any experience setting goals for yourself?

- Can you tell me more?
- Did you meet or achieve the goals you set for yourself in the group?
- If yes, can you tell me more?
- If no, what got in the way? What can the facilitators do to support you in meeting/achieving your goals?

5. After *M&C-Y* participation, how do you feel about your ability to set goals and meet them?

- Can you tell me more?
- Can you share an example?

**Active Rehabilitation**

6. Another important component of *M&C-Y* is physical activity/active rehab. Can you share with me how you felt about this part of the program?

- Can you tell me more?

7. How did moving your body make you feel?

- Did moving your body change how you felt about yourself?
- Did moving your body change how you felt about your ability to exercise?
- Have you noticed any difference in your ability to exercise or your daily routines after joining *M&C-Y?*
- Can you tell me more?

8. Can you tell me a little bit about what it felt like to do physical activity with a group?

- Would you say that you felt comfortable doing physical activity in a group? How so, or how not?
- What role did the facilitators play in helping you feel comfortable doing physical activity in a group setting?

**Group Dynamics**

9. Can you tell me what it was like to interact with other youth who have similar experiences as you?

- How did you feel meeting other youth in the group and sharing your experience?
- Was this your first time interacting with other youth experiencing concussion symptoms? If not, how was this experience the same or different from your past experiences?

10. Would you say you felt comfortable sharing your experience with the group?

- If yes, what made you feel comfortable?
- If no, what got in the way? What could the facilitators do better to make youth feel more comfortable in the group?

**Effect on day-to-day life**

11. Can you describe to me how participating in *M&C-Y* has had an impact on you?

- Can you tell me a little more about that?
- Can you share some examples of that?
- Has your perception of your injury (way you think about injury) changed after program participation? In what way?
- Has *M&C-Y* had any other impacts on your day-to-day life?

12. How do you think your participation in *M&C-Y* impacted your family members, like your parents or siblings?

- Can you tell me a little more about that?
- Can you share some examples of that?

***13. Only ask if caregivers participated in M&C-C****:* From your perspective, how do you believe *M&C-C* has had an impact on your parent?

- Do you think that your parent participating in *M&C-C* has helped them? If yes: In what way? If not, can you tell me more?
- Can you share some examples of that?

**Reflections & Future Improvements**

14. Thinking back to earlier in in the interview, we talked about the reasons you joined *M&C-Y*. Do you feel the intervention met your needs and expectations?

- If not, in what ways did it not meet your expectations?
- If so, can you provide some examples?

15. If we were to run *M&C-Y* again, what do you think we should keep in mind?

- Can you share any specific examples of things we might change?
- Can you share any specific examples of things we might keep the same?

**Caregiver Interview Guide**

Through this interview, I hope to learn about your experience with the Move&Connect-Caregiver program. The questions will be open-ended to allow you the chance to raise the issues that you feel are important. You do not have to answer any question you feel uncomfortable with, and you can stop the interview at any time. You can share as much or as little as you like. Your responses will be confidential and anonymized in any findings we may publish. Quotes from the interviews may be used, but it will not be possible to identify their source. Your participation and responses will not affect the care your child receives at Holland Bloorview Kids Rehabilitation Hospital.

Please note the interview will be audio recorded so that the research team can keep a record of the interaction. The recording will be deleted following transcription and won’t be used for any purpose other than the ones outlined. This record will let the team review and analyze your interview responses along with feedback from other study participants. And, I will be happy to answer any questions. Do you have any questions for me before we begin?

**General Questions**

1. Can you tell me what it was like to participate in *M&C-C?*

- Can you expand on what you did/didn’t like about that aspect of the program?

2. What made you decide to join *M&C-C*?? What did you hope to get out of *M&C-C*?

- Did you have any hesitations about joining *M&C-C*? If so, how were they overcome?

3. What made you come back to *M&C-C* each week?

- Was there anything in particular that supported/encouraged you to stay engaged with *M&C-C*?

**Group Dynamics**

4. Can you tell me what it was like to interact with other parents whose children are also experiencing symptoms of concussion?

- Was this your first time interacting with other parents of children experiencing concussion symptoms? If not, how was this experience the same or different from your past experiences?
- How did you feel sharing your experiences with the group?
- Did hearing other caregivers’ stories help you reflect on your own experience with your child’s injury? Can you tell me more about that?

**Effect on day-to-day life**

5. Can you describe to me how participating in *M&C-C* has had an impact on you as a parent of a child experiencing concussion?

- Can you tell me a little more about that?
- Can you share some examples of that?

***6. If their child participated in M&C-Youth:*** I’m interested in understanding more about your child’s experience with *M&C-Y*. From your perspective, how do you believe *M&C-Y* has had an impact on your child?

- Can you tell me a little more about that?
- Can you share some examples of that?

7. We’ve just discussed how *M&C-C* has had an impact on you as a parent, and how you believe *M&C-Y* has had an impact on your child. Now, I am interested in learning about how *M&C-C* has, or has not, affected you and your child together. For example, has *M&C-C* had any impact on your interactions and relationship with your child?

- Have the ways in which you support your child changed? How so?
- Has your perception of your child’s injury changed after program participation? In what way?
- Did you notice any changes in how you communicate with your child? How so?
- Has *M&C-C* had any other impacts on your day-to-day life?

**Ripple Effect Diagram**

This is the ripple effect diagram you drew for us after the end of the first session [*show the participant their ripple effect diagram that they submitted to the team*]:

8. Can you please explain your ripple effect diagram to me?

9. I’m curious to know how this ripple effect might be the same or different now that M&C has ended. Can I ask you to take a minute to draw your diagram again?

- I notice that this aspect of the ripple effect is the same as before. Can you please explain this similarity to me?
- I notice that this aspect of the ripple effect is different than before. Can you please explain this difference to me?
- Is there anything else about the ripple effect that you would like to share with me?

**Reflections & Future Improvements**

10. Thinking back to earlier in in the interview, we talked about the reasons you joined *M&C-C*. Do you feel the intervention met your needs and expectations?

- If not, in what ways did it not meet your expectations?
- If so, can you provide some examples?

11. If we were to run *M&C-C* again, what do you think we should keep in mind?

- Can you share any specific examples of things we might change?
- Can you share any specific examples of things we might keep the same?
