# Supplementary material for: Delivering Virtual Group Interventions for Youth With Persisting Symptoms After Concussion (Move&Connect-Youth) and Their Caregivers (Move&Connect-Caregivers): Fidelity and Engagement Evaluation
Source: JMIR Rehabil Assist Technol. 2026 Sep 21;13:e88771. doi: 10.2196/88771 (PMC13592660; doi:10.2196/88771)
Supplement: Supplementary file 3 [file rehab-v13-e88771-s002.docx]

**Supplementary Material**

Detailed clinician delivery checklist results and group engagement scores for *M&C-Y* and *M&C-C*. Each of the two clinician facilitators completed the clinician delivery checklist weekly (i.e. a total of six entries per clinician per group). Group engagement scores were scored out of three. The engagement rating was developed by the research team, and the scale’s reliability and validity have not been established.

|  | **Move&Connect-Youth** | | | | | | | **Move&Connect-Caregivers** | | | | | | |
| --- | --- | --- | --- | --- | --- | --- | --- | --- | --- | --- | --- | --- | --- | --- |
|  | **Discussion** | | **Exercise** | | **Goal Setting** | | **Group Engagement Score** | **Educational Topics** | | **Activity** | | **Take home reflection (weeks 2-6 only)** | | **Group Engagement Score** |
|  | **C1** | **C2** | **C1** | **C2** | **C1** | **C2** |  | **C1** | **C2** | **C1** | **C2** | **C1** | **C2** |  |
| **1** | 6/6 | 6/6 | 6/6 | 6/6 | 6/6 | 6/6 | 2.91 | 6/6 | 6/6 | 5/6 | 4/6 | 5/5 | 5/5 | 2.92 |
| **2** | 6/6 | 6/6 | 6/6 | 6/6 | 6/6 | 6/6 | 2.66 | 6/6 | 5/5  ** | 6/6 | 5/5** | 5/5 | 4/4  ** | 2.84 |
| **3** | 5/6  * | 5/6* | 5/6  * | 5/6  * | 5/6* | 5/6* | 2.93 | 5/5  * | 4/5  * | 5/5  * | 5/5* | 2/4* | 2/4  * | 2.90 |
| **4** | 6/6 | 6/6 | 6/6 | 6/6 | 6/6 | 6/6 | 3.00 | 5/6 | 6/6 | 5/6 | 6/6 | 4/5 | 4/5 | 2.50 |
| **5** | 6/6 | 6/6 | 6/6 | 6/6 | 6/6 | 6/6 | 2.67 | 6/6 | 6/6 | 6/6 | 6/6 | 4/5 | 3/5 | 2.58 |
| **6** | 6/6 | 6/6 | 6/6 | 6/6 | 6/6 | 6/6 | 2.77 | 6/6 | 6/6 | 6/6 | 6/6 | 5/5 | 5/5 | 2.72 |
| **7** | 6/6 | 6/6 | 6/6 | 6/6 | 6/6 | 5/6 | 2.83 | 6/6 | 6/6 | 6/6 | 6/6 | 5/5 | 4/5  **** | 2.96 |
| **8** | 4/5  ** | 5/6 | 4/5  ** | 5/6 | 5/5  ** | 6/6 | 2.78 | 5/5  * | 5/5  * | 5/5  * | 5/5* | 4/4* | 4/4  * | 3.00 |
| **9** | 6/6 | 6/6 | 3/6  *** | 4/6  *** | 5/6  *** | 5/6*** | 2.43 | 5/5  ** | 6/6 | 5/5  ** | 6/6 | 3/5** | 4/5 | 2.78 |
| **10** | 6/6 | 6/6 | 6/6 | 6/6 | 6/6 | 6/6 | 2.38 |  |  |  |  |  |  |  |

C1 = clinician one; C2 = clinician two; *one session canceled and not completed; **clinician absent from session due to illness; *** one session cancelled due to low attendance, but discussion added to following week; ****clinician data missing from part of checklist
